# Relationships between human tibia diaphysis shape and experimental injury outcomes

**DOI:** 10.1111/1556-4029.70381

**Published:** 2026-06-15

**Authors:** Christopher M. Goden, Amanda M. Agnew, Angela L. Harden

**Affiliations:** ^1^ Injury Biomechanics Research Center The Ohio State University School of Health and Rehabilitation Sciences Columbus Ohio USA

**Keywords:** blunt trauma, experimental loading, forensic anthropology, skeletal trauma analysis, tibia shape, virtual anthropology

## Abstract

Interpretation is central to skeletal trauma analysis; however, the influence of intrinsic skeletal variation, such as long bone shape, on injury is not fully understood. While previous studies have emphasized extrinsic variables such as loading rate, direction, and number of impacts, this study investigates whether diaphyseal shape variation of the tibia contributes to differential injury outcomes sustained under standardized blunt trauma experiments. Sixty adult human tibiae were subjected to controlled four‐point bending tests in a lateral‐medial direction at 6 m/s, and resulting fractures were analyzed by count and overall injury complexity. Diaphyseal surface morphology was quantified as continuous shape traits represented by unscaled and scaled principal components to capture size‐dependent and shape‐only variation, respectively. Multinomial logistic regression models identified shape‐injury associations and examined the moderating effects of age and sex. Multiple shape traits, particularly those involving anterior crest morphology and diaphyseal curvature, were significantly associated with fracture count and injury complexity. Notably, low‐variance traits also influenced outcomes, suggesting individual morphology may modulate injury outcomes. Sex‐ and age‐based interactions further indicate that similar morphologies may result in different outcomes between males or females and across adult age groups. Compared to fracture count, injury complexity produced more interpretable and biologically meaningful results. Collectively, these results show injury outcomes vary with tibial morphology, even under identical loading conditions, suggesting shape may warrant consideration when interpreting trauma in forensic casework. Accounting for shape variation may improve interpretation reliability, avoid misinterpretation of extrinsic factors, and support the development of probabilistic frameworks in forensic reconstructions.

**Highlights**

- Three‐dimensional tibia diaphyseal shape influences injury outcomes in blunt impact experiments.
- Shape–fracture relationships may obfuscate interpretations of skeletal trauma.
- Age and sex modulate how shape affects experimental trauma, likely reflecting underlying biological differences.
- Bone morphology may warrant consideration when interpreting skeletal trauma.

## 1 INTRODUCTION

Interpretations derived from skeletal trauma analyses influence medicolegal investigations and routinely result in expert witness testimony [1, 2, 3, 4, 5]. Accordingly, methods of trauma documentation and interpretation must be transparent, validated, and defensible under legal scrutiny. Best practices recommend that skeletal trauma be documented and, when possible, extrinsic factors be inferred and reported [5, 6]. However, limited guidance exists to inform how intrinsic human variation, particularly global bone shape, affects bone's response to loading.

Injury outcomes, such as fracture pattern, count, and complexity, are routinely used in trauma analysis to interpret extrinsic event characteristics, such as loading direction, velocity, and minimum number of impacts [4, 5, 7, 8, 9, 10]. It is commonly assumed that the same element (e.g., the tibia), when subjected to comparable loading conditions across individuals, will produce similar injury patterns, thereby supporting inferences about extrinsic factors in forensic contexts [5, 7, 11]. However, experimental research consistently demonstrates substantial variation in fractures resulting from blunt loading, even when extrinsic boundary conditions (e.g., loading rate and direction) are held constant [12, 13, 14, 15, 16, 17, 18, 19]. This residual variability suggests intrinsic factors relating to the bone itself also contribute to injury outcomes. Measurements of cross‐sectional geometry that capture the amount and distribution of bone (e.g., cortical area, cortical thickness, and robustness) are linked to bone strength [20, 21, 22, 23, 24]. Multiple studies have reported significant variability in cross‐sectional geometry among individuals and skeletal elements [25, 26, 27, 28, 29], underscoring how variability in bone geometry and strength may influence differential responses to loading. While informative, cross‐sectional geometry captures only a subset of skeletal morphology and may not fully reflect contributions of three‐dimensional (3D) shape variation to mechanical behavior. Even so, it has been suggested that intrinsic individual‐level differences in bone geometry introduce variability without undermining the interpretive utility of overall fracture morphology [11].

Statistical shape modeling (SSM), which quantifies three‐dimensional (3D) surface geometry, has been used to differentiate healthy and pathological bone, classify bones into fractured versus non‐fractured groups, and to approximate whole bone shape when fragmented [30, 31, 32, 33, 34, 35]. Bone shape has also been linked to general bone quality and fracture susceptibility in osteoporotic elements [30, 33]. However, it is unknown whether 3D morphology can predict specific injury outcomes (i.e., fracture count and classification) under controlled blunt loading. Examining the relationship between injuries sustained in controlled trauma experiments and SSM‐derived morphological traits may help clarify the relative contributions of intrinsic morphology and extrinsic factors to injury outcomes. If 3D morphological variation meaningfully influences injury outcomes, additional caution may be warranted when using skeletal injuries to reconstruct traumatic events.

The objective of this study is to evaluate the contribution of individual diaphyseal shape variation to injury outcomes within an experimental trauma sample of human tibiae. Establishing shape–injury relationships will advance the fundamental understanding of fracture variability and strengthen the scientific basis of forensic trauma analysis by clarifying the role of intrinsic and extrinsic factors.

## 2 MATERIALS AND METHODS

### 2.1 Sample composition and blunt trauma experiments

Fresh (i.e., not embalmed) human tibiae (*n* = 60) from adult postmortem human subjects (PMHS) were ethically obtained through The Ohio State University Body Donation Program. All potential specimens were pre‐screened for preexisting trauma and pathology, and any specimen exhibiting trauma or pathology involving the tibia was excluded from the study. A deliberate attempt was made to select tibiae from equal numbers of female (*n* = 31; 68.2 ± 20.8 years) and male (*n* = 29; 59.4 ± 21.8 years) donors across adult age categories (Table 1). Sample distribution did not statistically differ by sex (Welch's *t*‐test, *t*[1.6] = 57.3, *p =* 0.12). Differences in sample distributions remained insignificant by sex when stratified by age‐categories provided in Table 1 (*χ* ^2^(3) = 2.16, *p* = 0.54). The tibia side was randomly selected to include left (*n* = 25) and right (*n* = 35) tibiae. Demographic information was limited to self‐ or next‐of‐kin‐reported race, and the sample lacked sufficient variation to support evaluation of population‐level effects. Each isolated tibia underwent high‐resolution CT imaging (Philips Ingenuity Digital PET/CT; 120 kV, 262 mAs, 0.335 mm in‐plane resolution).

**TABLE 1:** Sample frequency and demographic information.

Age category					
	Young (24–40 years)	Middle (41–60 years)	Old (61–80 years)	Elderly (81–102 years)	*n*
Females	4	7	11	9	31
Males	7	8	9	5	29
*n*	11	15	20	14	60

**FIGURE 1:**
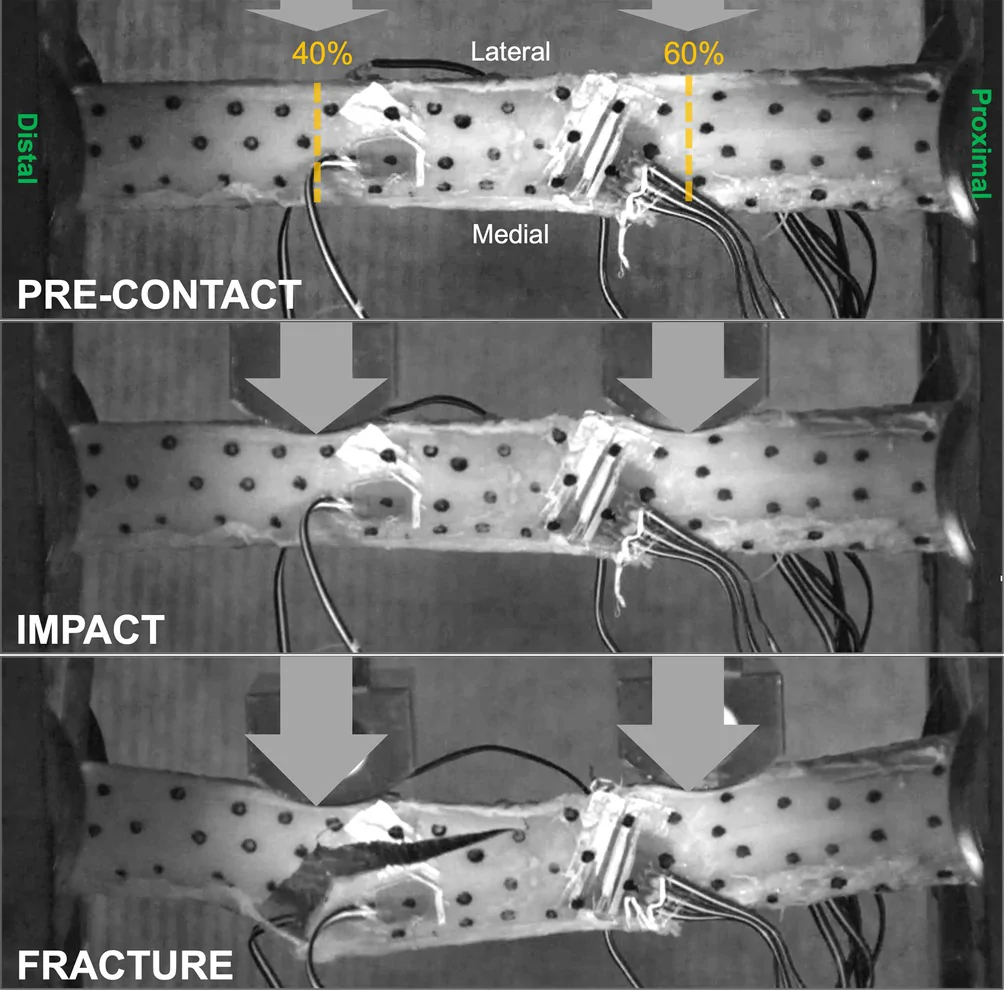
Stills from high‐speed video of 4‐point bending experiment, lateral‐medial direction at 6 m/s. Pre‐contact (top) shows bone in impact position (posterior surface facing camera, lateral surface superior, medial surface inferior, proximal on right, distal on left) with impactor target locations shown in yellow. Impact (middle) shows impactors contacting the 40% and 60% diaphyseal sites simultaneously. Fracture (bottom) shows propagation and the resulting fracture pattern. This experiment is a simplified scenario replicating a pedestrian–vehicle bumper impact conducted as part of a larger ongoing study.

Specimen preparation and experimental testing setup are detailed in [23], and only a brief overview is provided here. Soft tissue was removed while preserving the periosteum, and specimens were rigidly potted at the 20% (distal) and 80% (proximal) diaphyseal sites, calculated based on total bone length without the medial malleolus. Epiphyses were subsequently removed to facilitate proper positioning within the mechanical testing fixture. 3D laser scans of potted tibiae were acquired at 0.04 mm resolution in testing orientation (FARO Edge ScanArm, FARO Technologies, Lake Mary, FL) prior to impact. All tibiae were dynamically impacted simultaneously at the 40% and 60% diaphyseal sites via four‐point bending in a lateral‐medial direction at 6 m/s (Figure 1). Throughout the duration of the testing procedure, tibiae were kept wet to ensure specimens were biomechanically fresh and resulting fractures represented anthropologically perimortem trauma.

**FIGURE 2:**
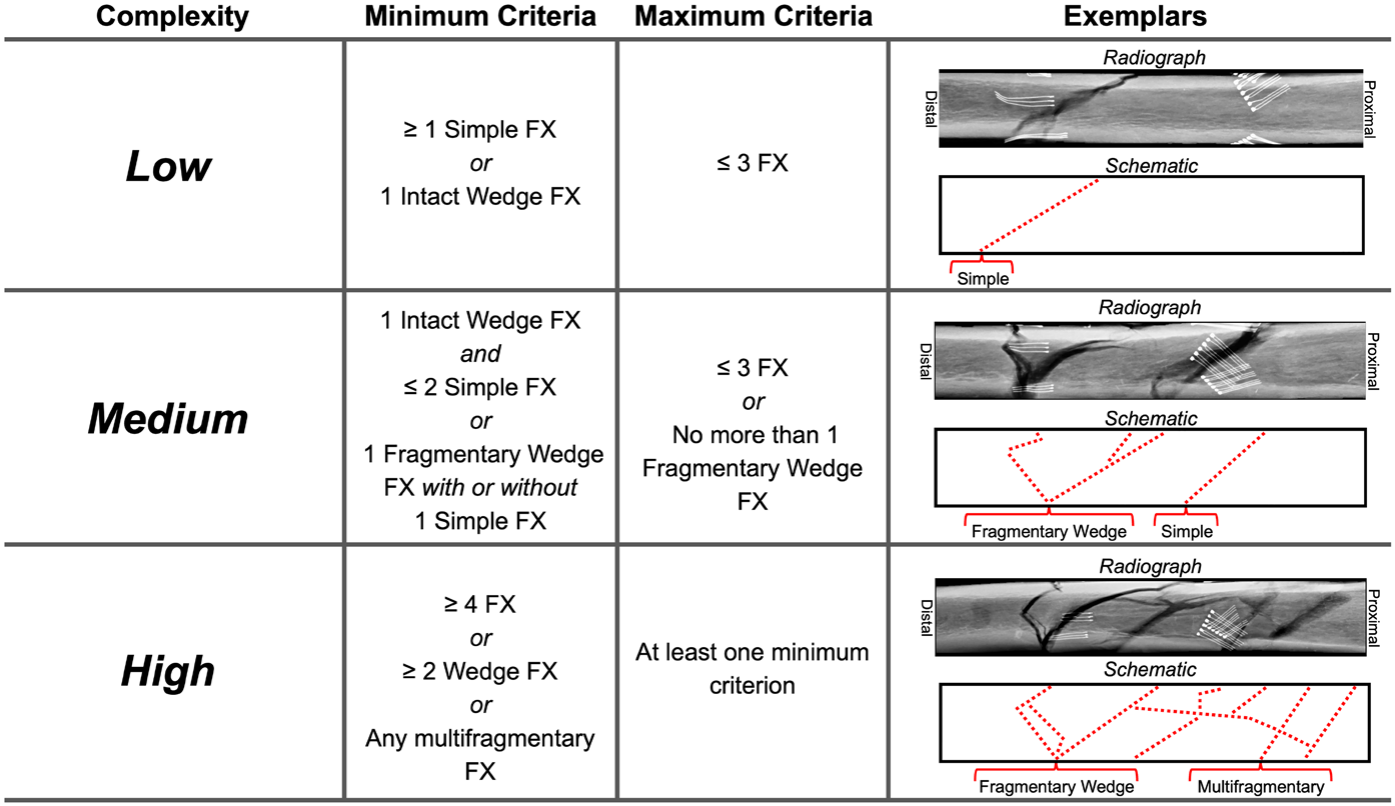
Minimum and maximum criteria for complexity tiers. Exemplar column shows radiographs (top) and simplified schematic (bottom), with example AO Foundation/Orthopaedic Trauma Association (AO/OTA) fracture classifications labeled on each schematic. Radiographs oriented viewing the posterior surface of the left tibia in impact position (lateral is superior, distal is left, proximal is right). Fracture denoted as “FX”.

After testing, radiographs were acquired (posterior to anterior projection [65 kVp, 3.2 mAs]) for visual representation, but the images were not utilized for fracture classification. Fractures were classified via gross examination of the physical specimens using the AO Foundation/Orthopaedic Trauma Association (AO/OTA) fracture and Dislocation Compendium, which categorizes each fracture hierarchically first by type (e.g., simple, wedge, or multifragmentary) and further assigns a group within each type (simple: transverse or oblique; wedge: intact or fragmentary; multifragmentary: intact segmental or multifragmentary segmental) [36]. Based on these classifications, the total number of discrete fractures per tibia was recorded (i.e., fracture count). One limitation of this classification system is that it does not assign severity to each fracture, and as a result, a single simple fracture and a single multifragmentary fracture are each counted as one fracture. Moreover, it has been suggested that singular fracture classifications may not be representative of the overall trauma present on a specimen [8]. To address these limitations, a novel whole‐element injury complexity index was developed to account for both the total fracture count and the observed AO/OTA fracture classifications present per tibia (Figure 2).

### 2.2 Quantification of shape traits

**FIGURE 3:**
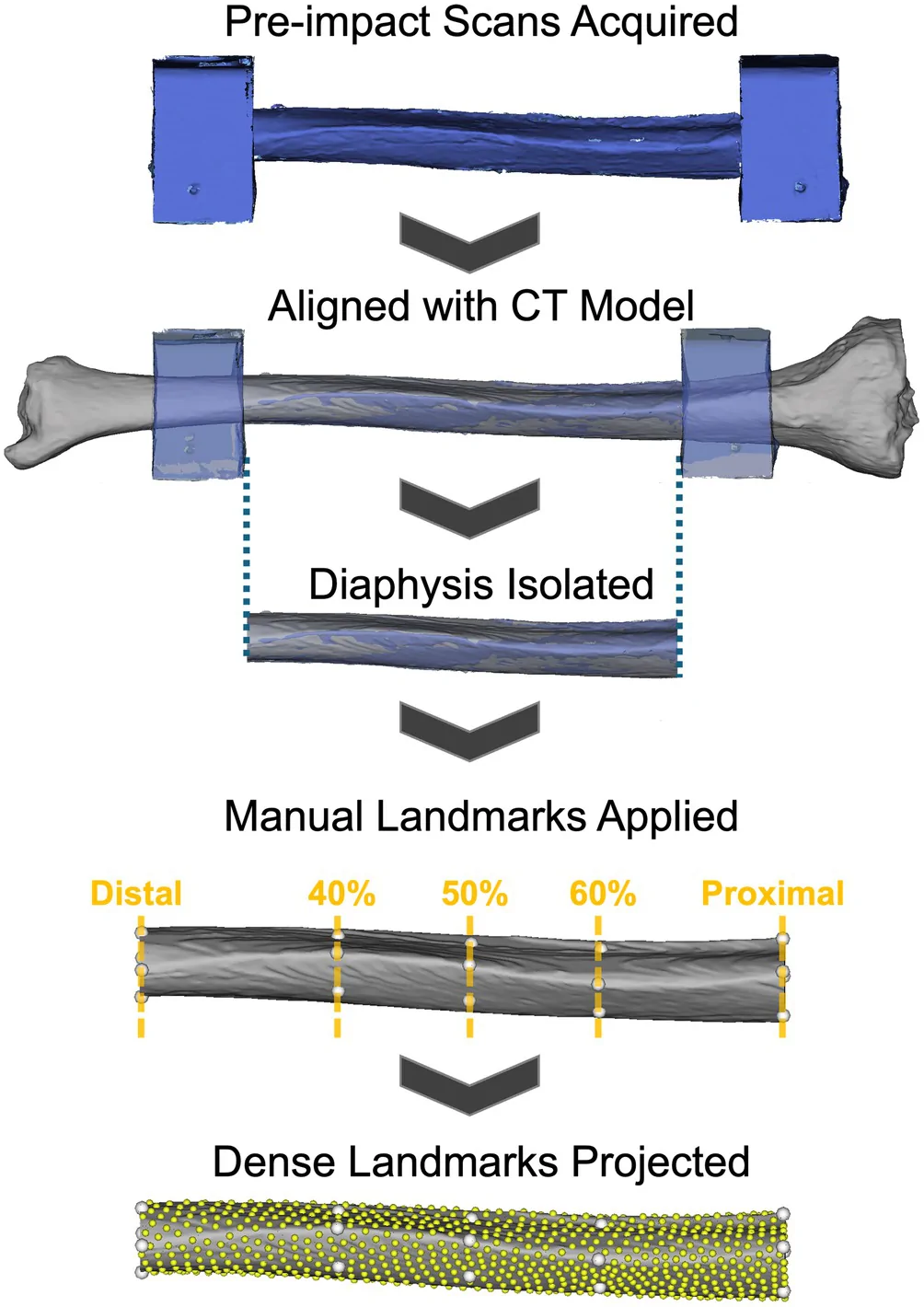
Workflow to establish an alignment and correspondence process to facilitate shape analysis of exact diaphyseal specimens subjected to bending experiments. Procedures were executed in 3DSlicer using modules included in the base software and SlicerMorph [37, 38, 39].

Pre‐test tibia CT scans were segmented in 3D Slicer (v 5.6.2) and rendered as watertight models to create meshes for shape analysis [37, 38]. Left tibiae were mirrored to appear as right to facilitate a single pooled sample for analysis. To ensure the shape analysis corresponded precisely to the experimental specimens, an alignment and registration protocol was developed (Figure 3). CT meshes were aligned to the pre‐test laser scans and cropped at the pots. This approach accurately isolated each diaphyseal mesh to match only the corresponding section of bone present for mechanical testing.

A dense‐landmark strategy was employed to quantify morphological variation across the sample. Twenty anatomical landmarks were manually placed on the anterior, posterior, medial, and lateral surfaces across five diaphyseal locations (20%, 40%, 50%, 60%, and 80% length). Using the SlicerMorph toolkits, a dense template of 1004 uniformly spaced pseudolandmarks was generated on the sample mean and projected onto each specimen via thin‐plate spline deformation [39, 40]. Final dense‐landmark positions were optimized relative to anatomical landmarks to facilitate surface correspondence across meshes [39, 40, 41].

To isolate specific morphological features for comparison with injury outcomes, generalized procrustes analysis (GPA) and principal component analysis (PCA) were performed under two conditions: (I) Unscaled principal components (UPCs): where residual translation and rotation differences were removed, but original size was preserved to capture the combined influence of size and shape, and (II) Scaled principal components (SPCs): where data were normalized by centroid size to isolate “pure” shape traits independent of bone scale. The first 10 PCs from both configurations were retained as continuous variables (shape traits) to explore relationships with injury outcomes.

### 2.3 Statistical analysis

Statistical analyses were performed to explore relationships between tibial morphology and injury outcomes and to determine whether covariates of sex and age influenced these relationships. To reduce dimensionality, analyses were confined to principal components (PCs) 1–10 for UPCs and SPCs. Because centroid‐size normalization redistributes variance and can arbitrarily alter PC rank and polarity [42], UPC and SPC datasets were each independently tested as predictors. Pearson's Correlation (*r*) was used to assess associations between UPCs and SPCs.

Multinomial logistic regressions were used to model the influence of shape on two categorical injury outcomes: fracture count (one [1], two [2], or three or more [3+]) and injury complexity (low, medium, or high). To comprehensively explore shape‐injury relationships, each PC was evaluated in unadjusted (PC only), covariate‐adjusted (sex or age), and interaction models (sex × PC or age × PC). Unadjusted models evaluated direct relationships between each shape trait and outcome variable, while covariate‐adjusted models accounted for the potential influence of sex and age (numeric and categorical). Interaction models were then employed to determine if sex or age moderated shape‐injury relationships. For categorical sex effect models, the interaction term estimated differences in outcomes for males relative to females. For age covariate and interaction tests, age groups were combined to maximize sample sizes into younger (24–60 years) and older (61+ years), with the interaction term estimating differences in outcomes for the older group relative to the younger group. For numeric age models, effects were modeled at 24 and 50 years, to represent the tibia from the youngest age in the sample and the age at which age‐associated bone loss may begin to affect biomechanical responses, respectively [43]. Odds ratios (OR) were used to quantify how the odds of each outcome changed with increasing PC scores, and predicted probabilities were derived from the positive and negative extremes of each PC to aid the interpretation of shape effects. Statistical analyses were conducted in R using the *multinom* function in the *nnet* package [44, 45].

## 3 RESULTS

### 3.1 Injury outcomes

**FIGURE 4:**
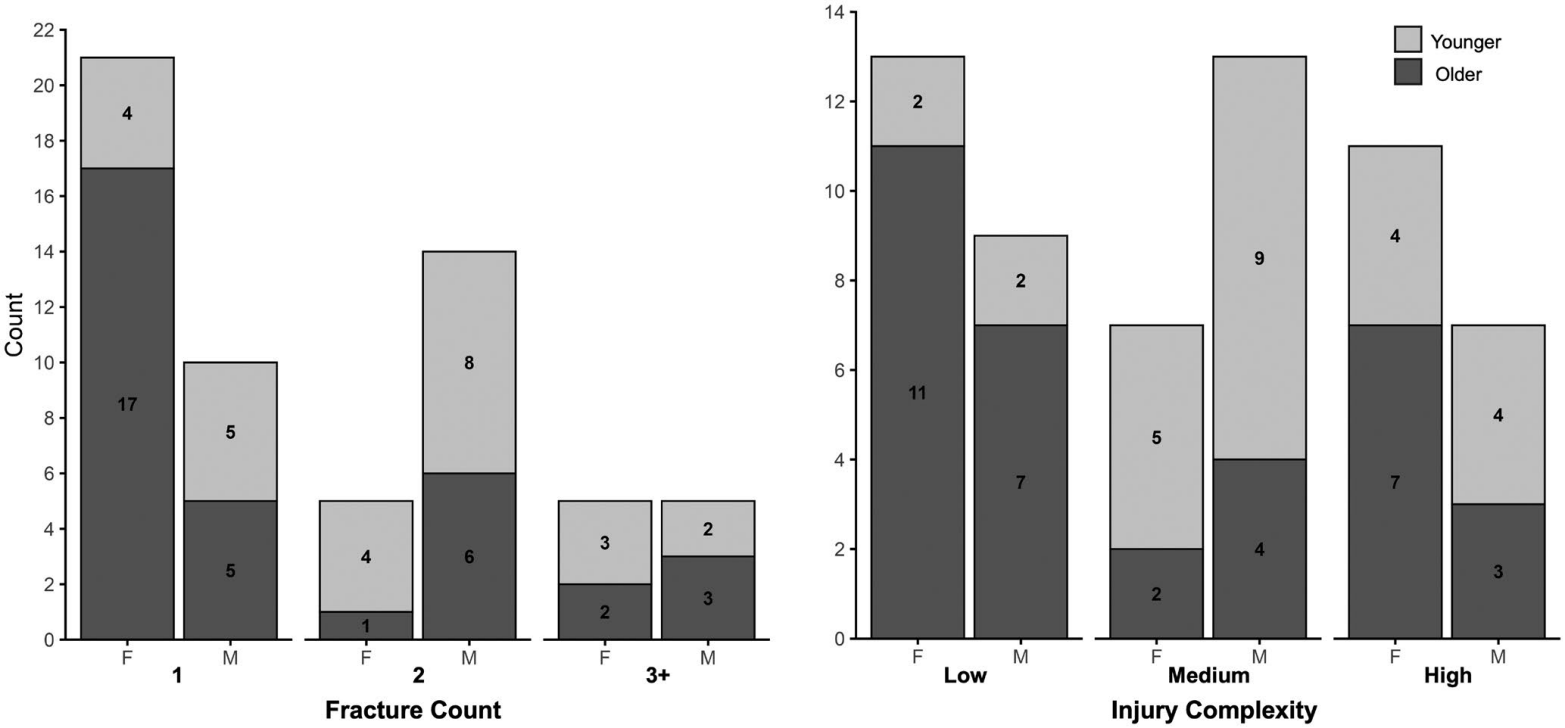
Bar charts with fracture count (left) and injury complexity (right) frequencies, by sex (females [F], males [M]) and age category.

Fracture count and injury complexity varied across all tibiae and when grouped by age and sex (Figure 4). Frequencies of fracture count per tibia were highest for 1 fracture (*n* = 31), followed by 2 fractures (*n* = 19), 3 fractures (*n* = 8), and 4 fractures (*n* = 2). Due to low frequency, tibiae with 3 and 4 fractures were collapsed to form a 3+ fracture group (*n* = 10). Tibiae from older adults exhibited a notably higher incidence of single fractures (*n* = 22), with the majority of those occurring in females (*n* = 17). Frequencies of injury complexity were more evenly distributed across the entire sample (low *n* = 22, medium *n* = 20, high *n* = 18). Tibiae with low and high injury complexity occurred more frequently in females (*n* = 13 and 11, respectively), whereas males exhibited the highest proportion of medium injury complexity (*n* = 13). Complexity also varied by age: over 50% of the tibiae in the older group had low complexity outcomes (*n* = 18) compared to the younger group (*n* = 4). In contrast, medium complexity outcomes were more frequent among the younger group (*n* = 14) than the older (*n* = 6).

### 3.2 Shape traits

For unscaled diaphyseal shape traits, the first ten PCs explained 98.3% of the total variation in the sample (Table 2). The first ten SPCs explained 88.2% of total variation in the scaled dataset, and dominant trait shapes were largely analogous to the unscaled data, though variance was redistributed across axes, and multiple modes that were the positive extreme in the unscaled dataset became the negative extreme in the unscaled dataset. Correlation analyses between UPCs and SPCs clarified these trends and demonstrated strong overlap in shape data (Table 2). Seven PC pairs exhibited very strong absolute correlations (*r* = 0.80–0.99), and two pairs showed strong correlations (*r* = 0.60–0.79), indicating that most shape modes were conserved across scaling conditions. SPC10 was uncorrelated with any other PC, supporting its interpretation as a distinct shape feature. Differences in sign between matched UPC and SPC eigenvectors reflected expected mathematical artifacts of size normalization, which alters the covariance structure and can invert polarity without changing morphological meaning. Detailed descriptions of the dominant modes of variation for each trait captured are reported in Table 2.

**TABLE 2:** Summary of dominant modes of shape variation observed at the minimum and maximum trait extremes.

Dominant traits	Minimum shape	Maximum shape	UPC (variance)	SPC (variance)	Correlation (*r*)
Global length	Short	Long	UPC1 (88.0%)	–	–
Anterior crest curvature and bowing	Proximally oriented curvature and anteriorly bowed diaphysis	Distally oriented curvature and straight diaphysis	UPC2 (3.8%)	SPC1 (27.8%)	0.99
Global width	Wide	Narrow	UPC3 (2.6%)	SPC2 (25.0%)	−0.87
Crest sharpness and lateral concavity	Blunt anterior crest with convex lateral surface	Sharp, hooked anterior crest with concave lateral surface	UPC4 (1.1%)	SPC3 (10.5%)	0.77
Proximal breadth and mass orientation	Narrow proximal breadth with anterior bowing and laterally oriented mass	Wide proximal breadth with a straight diaphysis and medially oriented mass	UPC5 (0.8%)	SPC4 (6.2%)	0.96
Diaphyseal circularity	Circular diaphysis	Triangular diaphysis	UPC6 (0.6%)	SPC6 (4.3%)	−0.94
Anterior crest sharpness and medial bowing	Sharp anterior crest with straight diaphysis	Blunt anterior crest with medially bowed diaphysis	UPC7 (0.5%)	SPC7 (3.7%)	−0.98
Proximal and midshaft tapering	Tapered proximal end with wide midshaft	Broad proximal end with thinned midshaft	UPC8 (0.4%)	SPC5 (5.3%)	−0.64
Interosseous crest projection and medial surface concavity	Protruding interosseous crest with convex medial surface	Smooth Interosseous crest with concave medial surface	UPC9 (0.3%)	SPC8 (2.1%)	0.97
Anterior crest shape	S‐shaped	Straight	UPC10 (0.2%)	SPC9 (1.9%)	−0.94
Distal torsion	Torsional distal half	Straight distal half	–	SPC10 (1.4%)	*–*

*Note*: Instances of negative correlation indicate that the same trait was identified between datasets; however, the sign of the trait (positive or negative) arbitrarily flips. As such, minimum shape and maximum shape descriptions are provided using the UPC dispositions and are the exact opposite of those for the correlated SPC.

Abbreviations: SPC, scaled principal component; UPC, unscaled principal component.

### 3.3 Fracture count multinomial logistic regressions

Only statistically significant results are summarized here, but complete multinomial results (including ORs with 95% confidence intervals, non‐significant results, and all associated raw predicted probabilities) are provided in the Supporting Information (Tables [Link], [Link]).

#### 3.3.1 Unadjusted models

Several unscaled shape traits (UPCs) were significantly associated with fracture count, specifically distinguishing between multiple and single fractures (Table S1). Higher UPC4 (*p =* 0.03) and UPC7 (*p* = 0.01) scores were associated with lower odds of sustaining 3+ versus 2 fractures (UPC4 OR = 0.95; UPC7 OR = 0.87). Predicted probabilities were consistent with these effects. For UPC4, the probability of 3+ fractures decreased from 49% at the minimum score to 5% at the maximum score, indicating a reduced likelihood of 3+ fractures among tibiae characterized by concave lateral surfaces and sharp, laterally oriented anterior crests relative to tibiae with convex lateral surfaces and blunt, medially oriented anterior crests. For UPC7, the probability of 3+ fractures decreased from 55% at the minimum score (sharp anterior crest) to <1% at the maximum score (blunt anterior crest), indicating that sharper anterior crests were associated with the highest fracture count (Table S1).

**FIGURE 5:**
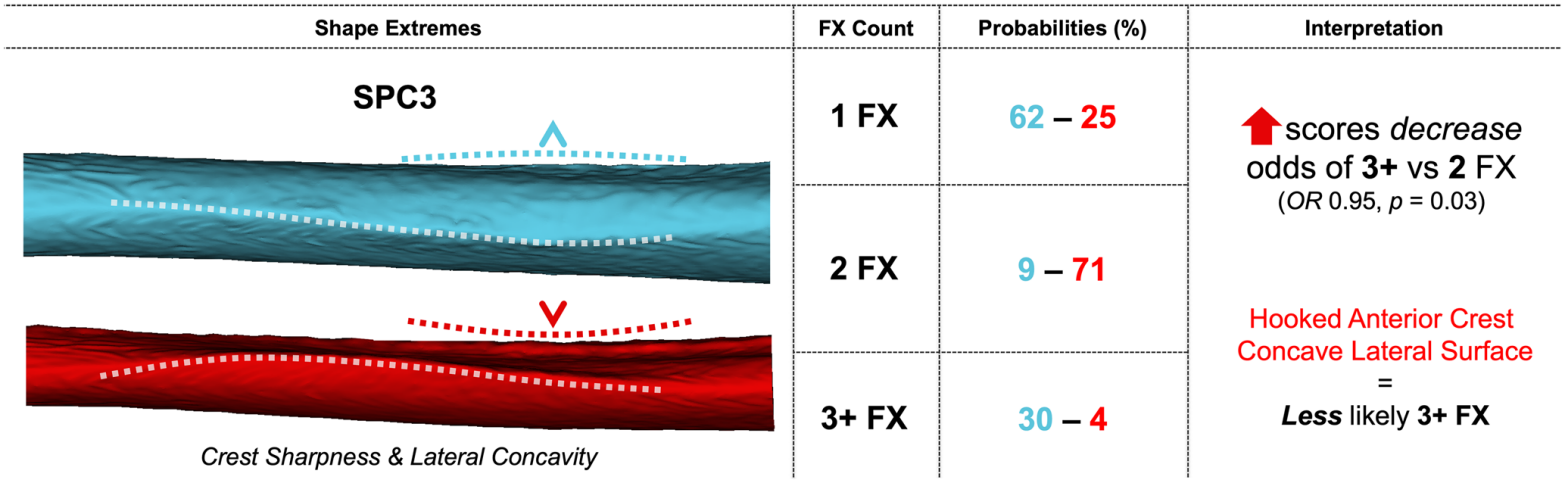
Influence of SPC3 (shown in anterior view) on fracture count. Shifting from a blunt anterior crest (blue [minimum score]) to a sharp, hooked crest (red [maximum score]) results in a substantial decrease in the predicted probability of 3+ fractures (30% vs. 4%). Arrows emphasize key morphological changes. Full results in Table S2.

Several scaled traits (SPCs) also exhibited significant relationships with fracture count (Table S2). SPC3 (*p* = 0.02) exhibited a bidirectional effect where higher scores increased the odds of 2 versus 1 fracture (OR = 2.07; predicted probabilities: 9% at minimum to 71% at maximum) but decreased odds of 3+ versus 2 fractures (OR = 0.38; predicted probabilities: 30% at minimum to 4% at maximum [Figure 5]). These trends were similar to those observed for UPC4, which was highly correlated with SPC3 (*r* = 0.77), though predicted probabilities differed slightly. Increases in SPC7 and SPC8 were significantly associated with fracture count (*p* ≤ 0.03), specifically between 3+ versus 2 (OR = 7.08) and 3+ versus 1 fracture (OR = 7.05) comparisons, respectively. Predicted probabilities indicated tibiae with blunt anterior crests and laterally shifted distal ends had <1% probability of 3+ fractures (minimum SPC7), compared with 41% for those with sharp anterior crests and medially rotated distal ends (maximum SPC7). Although UPC7 and SPC7 exhibited a strong correlation (*r* = −0.98), the polarity of their axes reversed, producing opposite numerical signs while describing the same shape trend. Higher SPC8 scores also corresponded to increased odds of sustaining 3+ fractures versus a single fracture. Tibiae characterized by smooth interosseous crests and concave medial surfaces (maximum SPC8) had a 40% probability of 3+ fractures, while those with convex medial surfaces (minimum SPC8) had only a 3%. In contrast, the probability of singular fractures along the SPC8 range was 77% for the minimum score compared to 26% at the maximum score.

#### 3.3.2 Covariate models

Including sex as a covariate preserved the associations between UPC4 and UPC7 and fracture count (Table S3). After accounting for differences between females and males, higher UPC4 (*p* = 0.04) and UPC7 (*p* = 0.01) scores continued to reduce the odds of 3+ versus 2 fractures (ORs ≤ 0.95). In addition, higher UPC7 scores increased the odds of 2 versus 1 fracture (*p* = 0.04; OR = 1.07). Predicted probabilities were comparable to those from the unadjusted models (Table S3). An additional association emerged for UPC9 (*p* = 0.04), where higher scores (less pronounced interosseous crest and concave medial surface) were associated with increased odds of 3+ versus 1 fracture (OR = 1.12).

For scaled traits, SPC7 and SPC8 remained significant (*p ≤* 0.03), with trends consistent with unadjusted analyses (Table S4). Sex effects were significant (*p* ≤ 0.04) across all models, indicating differences in fracture count between sexes after accounting for shape variation. Age‐adjusted models, whether categorical or continuous, yielded PC effects largely consistent with unadjusted analyses (Tables S5–S8). Age effects were significant for multiple UPCs (2–10) and SPCs (1, 3–6) in categorical models and across all numeric models. These findings suggest fracture count varied by age even after accounting for variation in diaphyseal shape.

#### 3.3.3 Interaction models

Interaction models were used to test whether relationships between tibial shape and fracture count differed by age or sex. No significant shape × sex interaction effects were identified (*p* > 0.05) (Tables S9 and S10), indicating males and females with comparable tibia morphology exhibited similar fracture counts. In contrast, multiple shape × age interactions were significant (*p* < 0.05), suggesting age moderated the influence of shape on fracture count (Tables S11–S14). For categorical age interactions, elevated UPC1 scores increased the odds of 3+ versus 1 fracture in the older group relative to the younger group (*p* < 0.01; OR = 1.02). Predicted probabilities reflected this pattern: longer tibiae (maximum UPC1) from older individuals were estimated to have a 70% probability of 3+ fractures, whereas long tibiae from younger individuals had only a 6% probability of 3+ fractures (Table S11). Elevated UPC3 scores decreased the odds of 2 versus 1 fracture in tibiae from older adults (*p* = 0.04; OR = 0.94), indicating a weaker tendency toward multiple fractures in narrow tibiae (maximum UPC3) when compared to tibiae from younger adults. A similar pattern was observed for the correlated scaled trait (SPC2; *r* = −0.87) (Tables S11 and S12). Additionally, SPC5 showed age‐modulated effects, with elevated scores associated with lower odds of 3+ versus 1 fracture in the older group. Predicted probabilities indicated a 2% probability of 3+ fractures at the maximum SPC5 score in older individuals compared with 44% in younger individuals (Table S12).

When age was modeled as a continuous interaction term (Tables S13 and S14), only the SPC6 (diaphyseal angularity) interaction term was significant (*p* = 0.03). Elevated SPC6 scores increased the odds of 3+ versus 1 fracture as age increased (OR = 1.43), suggesting the relationship between SPC6 and increased fracture count is attenuated with advancing age. When modeled at 24 years, tibiae with minimum SPC6 scores (triangular shape) were more likely to have 3+ fractures (96%) compared to those with high SPC6 scores (oval shape; <1%) (Table S14). When modeled at 50 years, this relationship persisted but was weakened, suggesting shape–fracture count relationships may become more variable with increasing age.

### 3.4 Injury complexity multinomial logistic regressions

#### 3.4.1 Unadjusted models

**FIGURE 6:**
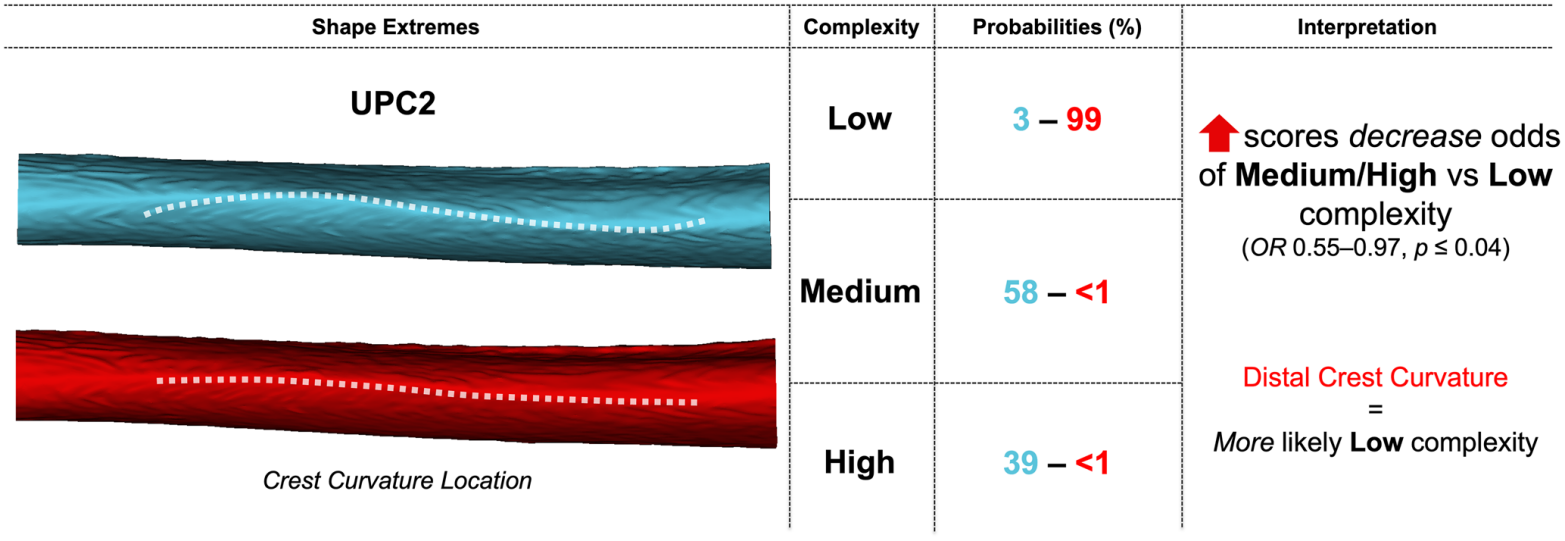
Influence of UPC2 (shown in anterior view) on injury complexity. Shifting from an anterior crest with proximally oriented curvature and anterior bowing (blue [minimum score]) to an anterior crest with distally oriented curvature and a straight diaphysis (red [maximum score]) results in a significant decrease in the predicted probability of high injury complexity (39% vs. <1%). Arrows emphasize morphological changes. Full results in Table S15.

Models for injury complexity identified relationships different from those associated with fracture count (Tables S15–S28). In the unscaled dataset, higher UPC2 scores (*p* ≤ 0.04; anterior crest curvature position) were associated with reduced odds of high versus low (OR = 0.97) and medium versus low complexity (OR = 0.96) (Table S15). Predicted probabilities clarified this trend (Figure 6): tibiae with distally oriented anterior crest curvature and a straight posterior border (maximum UPC2) exhibited <1% probability of high complexity, whereas tibiae with proximally oriented anterior crest curvature and anterior bowing (minimum UPC2) had a 39% probability. The analogous scaled component, SPC1 (*r* = 0.99), exhibited similar trends and nearly identical predicted probabilities (Table S16).

#### 3.4.2 Covariate models

When sex was included as a covariate, patterns for UPC2 and SPC1 remained significant and overall consistent with the original model, although p‐values and predicted probabilities shifted slightly (Tables S17 and S18). A new relationship was observed for SPC3 with maximum scores, reducing the odds of medium versus low complexity (*p* = 0.03; OR = 0.40). Associated predicted probabilities for low complexity increased from 25% (minimum SPC3) to nearly 70% (maximum SPC3), indicating pronounced, laterally hooked anterior crests combined with concave lateral surfaces corresponded to lower injury complexity. The covariate effect alone was significant only in models for UPC1, SPC1, and SPC3, indicating that after accounting for these traits, injury complexity differed between sexes (Tables S17 and S18).

**FIGURE 7:**
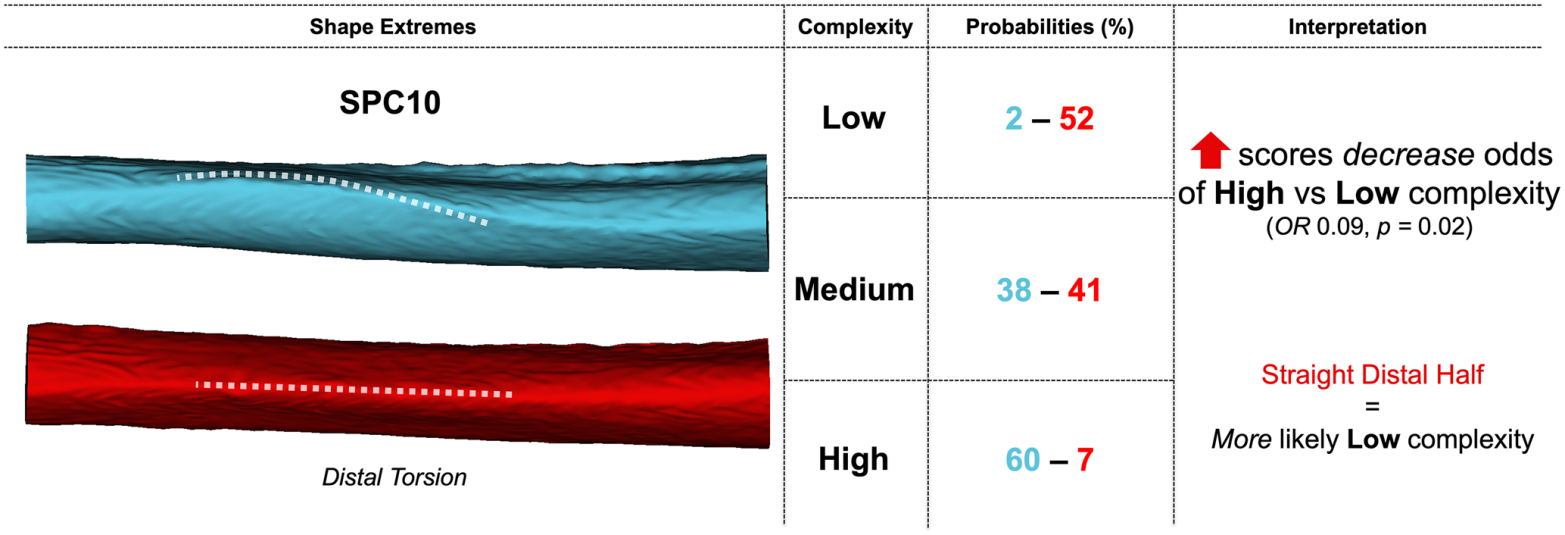
Influence of SPC10 (shown in anterior view) on injury complexity, controlling for categorical age. Shifting from a tibia with a torsional distal half (blue [minimum score]) to a straight distal half (red [maximum score]) results in a significant decrease in the predicted probability of high injury complexity (60% vs. 7%). The white dashed line emphasizes morphological changes. Full results in Table S20.

Including age as a covariate substantially altered results (Tables S19–S22). UPC2 and SPC1 were no longer significant (*p* > 0.05), while UPC4 and SPC10 (*p* < 0.04) became significant. Elevated UPC4 scores were associated with reduced odds of medium versus low complexity (OR = 0.95), with the predicted probability of medium complexity decreasing from 83% (minimum UPC4) to 30% (maximum UPC4) (Table S19). Predicted probability trends further suggested tibiae with sharp, laterally oriented anterior crests (maximum UPC4) exhibited relatively equal probabilities of low, medium, and high complexity (predicted probabilities = 30%–35%, each). For scaled data, models controlling for categorical age identified SPC10 as significant (*p* = 0.02), with higher scores reducing the odds of high versus low complexity (OR = 0.09) (Table S20). The predicted probability of high complexity decreased from 60% at minimum SPC10 (tibiae with distal torsion) to 7% at maximum SPC10 (tibiae with a straight distal half). These results suggest tibiae with distal torsion were likely to have more complex injuries (Figure 7). Models using continuous age yielded similar results (Tables S21 and S22). Additionally, significant covariate effects for both categorical and numeric age (*p* ≤ 0.03) were identified across all models, indicating age influenced injury complexity independent of shape.

#### 3.4.3 Interaction models

**FIGURE 8:**
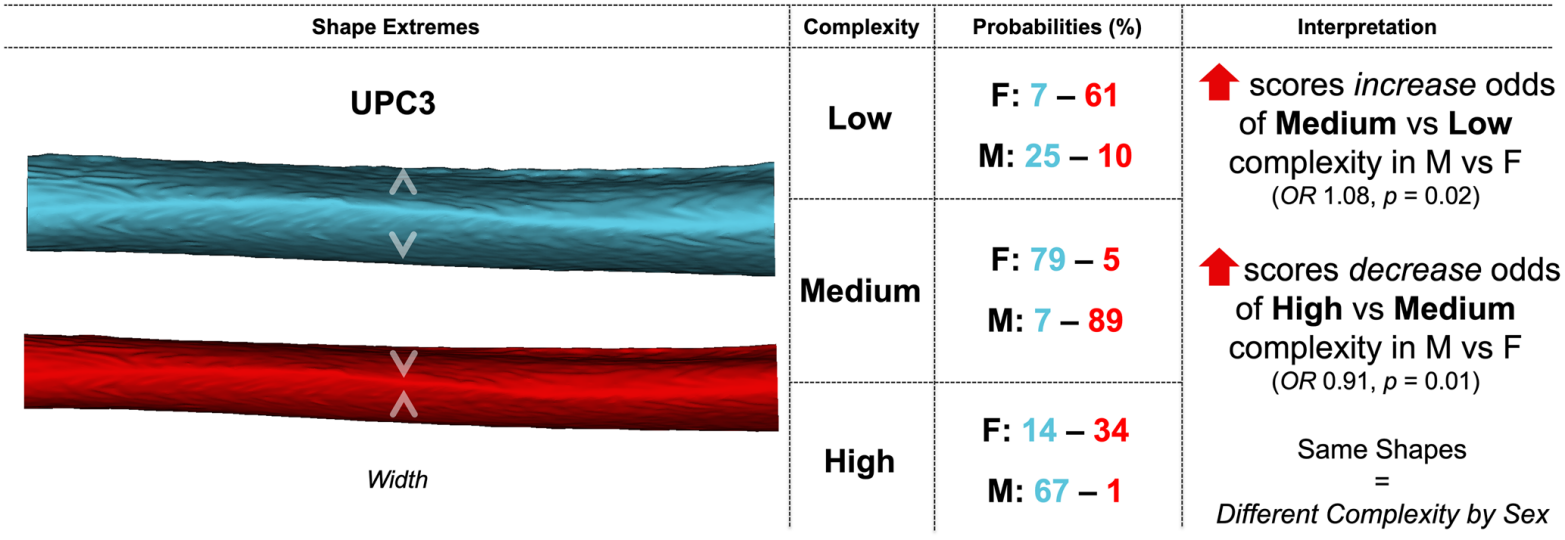
Sex interaction effect of UPC3 (shown in anterior view) on injury complexity. In wide tibiae (blue [minimum score]), females (F) were significantly more likely to sustain medium complexity injuries compared to males (M). In narrow tibiae (red [maximum score]), females were significantly less likely to sustain medium complexity injuries than males. Arrows emphasize morphological changes. Full results in Table S23.

As with fracture count, interaction models indicated that shape‐complexity relationships were not uniform across groups. Significant sex‐shape interactions were identified for UPC3 (*p* ≤ 0.02; tibia width), UPC5/SPC4 (*p* = 0.04; lateral or medial mass orientation, midshaft anterior bowing), UPC8 (*p* = 0.01; proximal tapering, midshaft thinness). Elevated UPC3 scores corresponded to higher odds of medium versus low complexity (OR = 1.08) and lower odds of high versus medium complexity (OR = 0.91) in tibiae from males compared to females (Tables S23 and S24). Predicted probabilities demonstrated narrow tibiae (maximum UPC3) had a 10% probability of low complexity in male tibiae versus 61% in female tibiae, while the same trait in male tibiae showed an 89% probability of medium complexity compared to 5% in female tibiae (Figure 8).

In tibiae from males, elevated UPC5 scores were associated with reduced odds of high compared to medium complexity (OR = 0.89), while higher UPC8 scores were associated with reduced odds of medium compared to low complexity (OR = 0.80). For UPC5, predicted probabilities support tibiae with anterior bowing at the midshaft (minimum score) exhibited a 7% probability of high complexity in tibiae from males, compared to 71% for the same trait in tibiae from females (Table S23). Trends were similar in the correlated SPC4 (*r =* 0.96) (Table S24). For maximum UPC8 (broad proximal ends with a narrowed midshaft), tibiae from males were associated with a 13% probability for medium complexity relative to 96% in tibiae from females (Tables S23 and S24). Sex interaction model results indicate that tibiae with comparable morphologies subjected to the same loading conditions exhibit different injury outcomes between sexes.

While PC effects for UPC2 remained significant, categorical age × shape interactions did not reach significance (Tables S25 and S26). Significant age × shape interactions demonstrated that age modified the relationships between shape and injury complexity when age was modeled as a continuous variable (Tables S27 and S28). Increasing age amplified the effects of UPC2 and UPC5 (*p* ≤ 0.04), both of which were associated with higher odds of increased injury complexity relative to low (Table S27). UPC8 (*p* = 0.03) showed an inverse trend with lower odds of medium complexity compared to low with increasing age (OR = 0.97).

Specifically, UPC2 increased the odds of high and medium versus low (OR = 1.01), suggesting the same shape trait predicts higher injury complexity in tibiae as age in the sample increases. Similarly, higher UPC5 scores increased the odds of high versus medium complexity (OR = 1.03), while higher UPC8 scores decreased the odds of medium versus low complexity (OR = 0.97). Scaled components SPC1 and SPC4 displayed parallel patterns to their unscaled counterparts (UPC2 [*r* = 0.99] and UPC5 [*r* = 0.96]), though SPC4 additionally increased odds of high versus low complexity (*p =* 0.04; OR = 1.30). Predicted probabilities modeled at 24‐ and 50‐years reflected these trends (Tables S27 and S28). Collectively, age‐shape interaction results indicate that a trait predictive of one complexity category in tibiae from younger individuals may correspond to different injury complexities in older individuals.

## 4 DISCUSSION

A persistent challenge in skeletal trauma analysis is attributing variability in outcomes to extrinsic loading factors. Although extrinsic factors such as loading rate, direction, and number of impacts are well‐established contributors to fracture variability [4, 11], the present findings suggest intrinsic diaphyseal shape also contributes to injury outcomes and may complicate interpretive heuristics commonly applied in skeletal analysis. For example, multiple fractures observed on a tibia characterized by a blunt anterior crest and convex lateral surface (minimum UPC4) might be interpreted as evidence of multiple impacts. However, the present findings indicate that a single dynamic lateral impact to a tibia with this morphology can also produce multiple fractures (Table S1). Although interpretations of skeletal trauma integrate principles of bone biology and biomechanics [1, 2, 46, 47], results suggest intrinsic factors may also warrant consideration when evaluating trauma.

### 4.1 Diaphyseal shape analysis

Several higher‐order PCs were predictive of injury outcomes despite accounting for relatively low variance, particularly when considering unscaled data. While often overlooked in shape analyses in favor of the first few PCs that describe broad population‐level trends, these higher‐order components can capture subtle morphological changes and outliers [48]. Such traits likely reflect individualized morphology meaningful to injury outcomes and biomechanical response [49]. Moreover, previous research demonstrates that low‐variance traits are useful to differentiate pathologic from healthy bone, predict biomechanical response, and capture sexual dimorphism [31, 50, 51, 52]. Even subtle shape differences may alter how bone responds under dynamic loading and, in forensic and injury biomechanics settings, might help explain why ostensibly similar bones yield dissimilar injuries. It is also worth noting that while modeling PCs separately clarified trait‐specific interpretations in this study, the co‐occurrence of traits was not evaluated. Anatomical traits do not exist in isolation, and their interactions may compound or mitigate injury outcomes. Future work should leverage advanced multivariate and machine learning approaches to comprehensively investigate the collective influence of unique trait combinations.

### 4.2 Injury outcomes and the influence of shape

Injury outcomes were assessed using fracture count and a novel injury complexity scheme integrating fracture classification and multiplicity. While fracture count models yielded significant associations with shape, interpretability was complicated. Conceptually, under the count models, different fracture types are classified equivalently, which conflates multiplicity with severity (e.g., transverse versus multifragmentary). Such oversight likely contributed to ambiguous model predictions. For example, in the unadjusted fracture count model, tibiae with blunt, medially oriented anterior crests (low UPC4) showed ambiguous probabilities for 1 or 3+ fractures relative to 2 fractures (Table S1).

In contrast, injury complexity produced more consistent and interpretable effects across models. Results highlight that injury complexity provides a more comprehensive measure of total bone injury. For example, this study found that tibiae with proximally oriented anterior crest curvature and anterior bowing (minimum UPC2/SPC1) were more likely to be associated with higher complexity outcomes, compared to those with more distally oriented crest curvature and minimal bowing (Figure 6). One possible explanation is that more curvature may serve as a stress concentrator, facilitating fracture initiation with unstable propagation and branching events, increasing fragmentation. In contrast, a more uniform diaphyseal geometry may distribute energy more evenly and fail along a more constrained trajectory, producing a less complex injury.

Covariate‐adjusted models similarly identified shape configurations associated with reduced injury complexity. Tibiae with sharp, laterally hooked crests and pronounced lateral concavity (high UPC4/SPC3) were more likely to sustain low‐complexity outcomes. Likewise, higher SPC10 scores (reflecting a straighter, less torsional distal half) were less likely to be associated with high‐complexity outcomes. These morphologies may influence how lateral loading is distributed along the shaft. For instance, torsional contours may redirect stress pathways, potentially producing different injury outcomes even under comparable loading. While speculative, prior work supports the notion that cross‐sectional geometry and loading orientation affect tibial strain distribution [8, 53, 54], making it plausible that external geometry also contributes to fracture propagation.

### 4.3 Interaction effects on fracture‐shape relationships

Demographic interactions modulated several shape‐injury relationships where comparable surface geometries yielded divergent outcomes between males and females. For instance, in wide tibiae (maximum UPC3), females were most likely to sustain low complexity outcomes, while males trended toward medium complexity (Table S23). Similar patterns were observed across several traits; however, they were most frequent in unscaled models (Table S23). This likely reflects known allometric differences in bone size and robustness that influence cross‐sectional geometry. These differences may involve variation in the distribution of cortical bone and associated changes in microstructural properties that cannot be captured by surface geometry alone [20, 55, 56].

Age introduced significant variability when considering interaction models. Interestingly, traits associated with low complexity in younger adults (e.g., UPC2/SPC1) became increasingly associated with higher complexity with increased age (Tables S25–S28). Likewise, higher UPC5/SPC4 increased the odds of high versus medium complexity with increased age, while UPC8 showed the opposite trend. Overall, predictive shape‐injury relationships became less stable with increasing age, which indicates biomechanical influence of global morphology may be attenuated by age‐related changes in bone quality. These patterns align with known age‐related changes in bone quality, including increased porosity, reduced collagen content, and decreased cortical area and thickness [14, 23, 28, 57], suggesting that relationships are not static and evolve with increasing age. While further research is necessary, it may be possible that comparable external morphologies yield divergent outcomes under similar loading due to sex or age‐related differences. As surface shape alone cannot fully capture the hierarchical and compositional intricacies of bone, future work should integrate cortical and trabecular morphometrics, histomorphometry, and finite element modeling to more comprehensively evaluate injury risk.

Nonetheless, although sex was included as a covariate, shape and sex are not entirely independent, given that sexual dimorphism is often expressed through shape [58, 59, 60, 61]. Adjusting for sex as a covariate statistically adjusts for sex‐related differences in a statistical model, allowing estimation of shape‐injury associations beyond those explained by sex. However, because sexual dimorphism is inherently expressed through shape, some shared variance remains, and the effects of sex and morphology cannot be fully disentangled, warranting further investigation with more advanced frameworks and larger sample sizes.

### 4.4 Implications for forensic science and methodological limitations

While promising, these results must be interpreted within the study's methodological constraints, and caution is warranted before applying them directly to forensic casework. This study used isolated human tibial diaphyses from a broad adult age range tested under highly controlled dynamic loading conditions; therefore, the findings may not generalize to global populations or reflect the full range of trauma scenarios encountered in forensic practice. As such, future work using more diverse samples may help clarify whether broader population‐level variation contributes to tibial diaphyseal shape and injury outcomes alongside size‐ and sex‐associated variation. At the same time, an important takeaway is that even under these simplified and tightly controlled conditions, substantial variance in injury outcomes remains difficult to explain. This highlights the complexity of fracture behavior and suggests that key factors influencing injury variability are not yet fully understood. Improving experimental biofidelity remains an important long‐term goal, but a necessary step is first identifying and clarifying the variables driving injury outcomes in controlled models. Once these relationships are better resolved, future work can more effectively extend to more complex, real‐world scenarios, including cadaveric studies with intact soft tissue and variable boundary conditions. In parallel, subject‐specific finite element modeling may help clarify how morphology interacts with loading conditions to produce differential fracture outcomes.

From a forensic perspective, these findings do not replace existing interpretive heuristics but suggest the value of situating them within a broader biological context. Although forensic anthropologists routinely ground their interpretations in skeletal biology and biomechanics [8, 46], the extent to which individual skeletal variation contributes to trauma outcomes remains incompletely characterized. Anatomical predispositions may influence injury patterns in ways that could otherwise be attributed to extrinsic loading conditions. Framing interpretations probabilistically acknowledges the potential influence of intrinsic bone morphology and material properties on observed outcomes. As the field continues to emphasize empirically supported and reproducible approaches, recognizing that fracture variability may reflect intrinsic skeletal characteristics further strengthens the evidentiary basis of expert opinion.

Ultimately, this work should be viewed as foundational for generating morphology‐based hypotheses to improve knowledge of shape‐injury behavior and to support eventual integration of quantitative morphology into trauma analysis. As such, observed associations require validation in future confirmatory research with more complex metrics. With continued development, this paradigm may progress to more nuanced, individualized, and biomechanically plausible forensic interpretations in research and applied settings.

## 5 CONCLUSION

Tibia shape exhibits multifaceted relationships with experimentally induced trauma resulting from a controlled blunt impact scenario. Multiple diaphyseal traits were predictive of different injury outcomes. Notably, several higher‐order PCs, despite accounting for small proportions of variance, exhibited significant associations with injury outcomes, indicating even subtle individualized differences in shape inform bony response. Interactions with sex and age highlight that similar external morphologies can lead to divergent outcomes, underscoring the need to better understand how demographic factors are related to bone shape and trauma. While shape analysis is not yet incorporated into trauma interpretation frameworks, these findings provide foundational evidence that warrants investigation of shape as a potential confounding variable in skeletal trauma analysis. As forensic anthropology continues to make advances toward probabilistic and evidence‐based methodologies, integrating bone shape into trauma research will provide a more robust empirical framework, potentially reducing error in interpretations and strengthening the credibility of expert testimony.

## FUNDING INFORMATION

Portions of this work were funded by the National Institute of Justice Research and Development in Forensic Science for Criminal Justice Purposes funding opportunity (award number 2019‐DU‐BX‐0040). The opinions, findings, and conclusions expressed in this publication are those of the authors and do not necessarily reflect the views of the National Institute of Justice.

Portions of this research were presented at the 78th Annual Scientific Conference of the American Academy of Forensic Sciences, February 9‐13, 2026, in New Orleans, LA.

## CONFLICT OF INTEREST STATEMENT

The authors have no conflicts of interest to report.

## Supporting information


Table S1.–S14.

Table S15.–S28.

## Data Availability

The data that support the findings of this study are available from the corresponding author upon reasonable request.
